# The Role of Silicon in Higher Plants under Salinity and Drought Stress

**DOI:** 10.3389/fpls.2016.01072

**Published:** 2016-07-18

**Authors:** Devrim Coskun, Dev T. Britto, Wayne Q. Huynh, Herbert J. Kronzucker

**Affiliations:** Department of Biological Sciences, Canadian Centre for World Hunger Research, University of Toronto, TorontoON, Canada

**Keywords:** silicon, salinity stress, drought stress, sodium toxicity, osmotic stress, apoplast, water transport, ion transport

## Abstract

Although deemed a “non-essential” mineral nutrient, silicon (Si) is clearly beneficial to plant growth and development, particularly under stress conditions, including salinity and drought. Here, we review recent research on the physiological, biochemical, and molecular mechanisms underlying Si-induced alleviation of osmotic and ionic stresses associated with salinity and drought. We distinguish between changes observed in the apoplast (*i.e.*, suberization, lignification, and silicification of the extracellular matrix; transpirational bypass flow of solutes and water), and those of the symplast (*i.e.*, transmembrane transport of solutes and water; gene expression; oxidative stress; metabolism), and discuss these features in the context of Si biogeochemistry and bioavailability in agricultural soils, evaluating the prospect of using Si fertilization to increase crop yield and stress tolerance under salinity and drought conditions.

## Introduction

Although appreciated by biologists for >150 years, the benefits of silicon (Si) to plants, particularly under stress, have been studied intensively only in recent decades. This is largely due to silicon’s “non-essential" designation by early plant nutritionists (*e.g.*, Sachs, 1860; Arnon and Stout, 1939; see also Epstein, 1999; Liang et al., 2015, for historical overview). Indeed, Si is not considered “essential" for higher plants, as they can fulfil their life cycles without it (Epstein, 1999; Liang et al., 2015). Nevertheless, Si is considered to be “quasi-essential" (Epstein and Bloom, 2005), due to the far-reaching benefits it confers on plants, including enhanced growth, yield and crop quality, photosynthesis, N_2_ fixation, particularly in response to abiotic and biotic stresses such as infectious disease, herbivory, gravity, metal toxicity, high and low temperature, UV radiation, nutrient deficiency and excess, drought, and salinity (for review, see Epstein, 1994, 1999, 2009; Richmond and Sussman, 2003; Ma, 2004; Liang et al., 2007, 2015; Cooke and Leishman, 2011; Guntzer et al., 2012; Van Bockhaven et al., 2013).

However, despite much recent research, the mechanisms underlying these effects are not well understood, although important new insights into the membrane transport of Si (Ma and Yamaji, 2006, 2015; *cf.* Exley, 2015), and the alleviatory role of Si in biotic stress (Ma, 2004; Van Bockhaven et al., 2013; Liang et al., 2015) have been gained. Mechanistic understanding of the role of Si in abiotic stress resistance, however, is relatively limited (Liang et al., 2015), but important avenues of research in salinity and drought contexts are emerging (for review, see Ma, 2004; Liang et al., 2007; Zhu and Gong, 2014). Salinity stress affects over 800 million hectares globally – up to a third of all agricultural land and nearly half of all irrigated land, which produces roughly a third of the world’s food (Zhu, 2001; Rengasamy, 2010). Drought stress, which shares many features with salinity stress (Munns, 2002), is even more pervasive and damaging to agricultural production, particularly in arid and semi-arid regions, which account for approximately 30% of the world’s land area (Boyer, 1982; Eneji et al., 2008; Farooq et al., 2009). Both problems are predicted to be aggravated by anthropogenic climate change (Yeo, 1999; Schmidhuber and Tubiello, 2007).

In this mini-review, we focus on current understanding (and gaps therein) of the mechanisms underlying silicon-induced alleviation of salinity and drought stress in higher plants, and will also discuss the feasibility of Si amendments in arid or saline agricultural fields. Where possible, we distinguish apoplast effects (*i.e.*, suberization, lignification, and silicification of the extracellular matrix; transpirational bypass flow of solutes and water), from those of the symplast (*i.e.*, transmembrane transport of solutes and water; gene expression; oxidative stress; metabolism), and consider where mechanistic overlaps exist.

## Si-Induced Changes to the Extracellular Matrix (Apoplast)

Silicon is well documented to strengthen cell walls and provide mechanical support for monocots and pteridophytes (much less is known about dicots), by enhancing suberization, lignification, and silicification (for a recent review, see Guerriero et al., 2016). Improved structural stability has been attributed to the binding of Si with cell-wall hemicellulose (He et al., 2013, 2015; Ma et al., 2015), which is clearly beneficial under water deficit. In addition, biosilicification in plants, involving the polymerization of silicic acid within the apoplast, leads to the formation of an amorphous silica barrier (Exley, 2015), which can help alleviate both biotic and abiotic stresses, hindering pathogen infection and the penetration of potential toxicants such as aluminum (Al), manganese (Mn), cadmium (Cd), zinc (Zn), and sodium (Na), into the symplast and/or transpiration stream (Rogalla and Römheld, 2002; Wang et al., 2004; Fauteux et al., 2005; Saqib et al., 2008; Ma et al., 2015; Guerriero et al., 2016). In roots of salt-sensitive and -tolerant wheat, for example, Si increased cell-wall binding of Na^+^ in the root while decreasing its transport to the shoot (Ahmad et al., 1992; Saqib et al., 2008; see also below); however, direct evidence of Na^+^ complexation by Si, which may underlie this potentially important salt-tolerance mechanism, is lacking.

Silicon has also been shown to promote Casparian band development in the root endodermis and exodermis (Fleck et al., 2011, 2015). For example, in rice, Si treatment resulted in enhanced suberization, and lignification of sclerenchyma, in these tissues (Fleck et al., 2011; *cf*. Suzuki et al., 2012), which coincided with reduced radial oxygen loss and oxidation power in the mature root (Fleck et al., 2011). Si also triggered the transcription of genes related to lignin and suberin synthesis (Fleck et al., 2011; see below). These components can form barriers to apoplastic Na^+^ transport in roots, correlating with higher salt tolerance in rice (Krishnamurthy et al., 2011). In particular, Si deposition in the endodermis is proposed to restrict Na^+^ transport along a “transpirational bypass” route from root to shoot in rice (Gong et al., 2006), as we shall now discuss.

## Transpirational Bypass Flow

**FIGURE 1:**
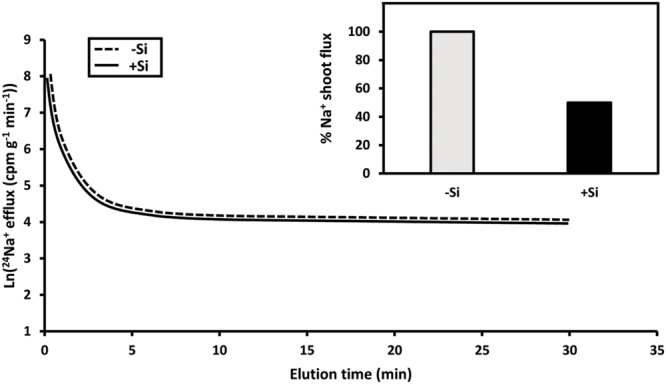
**Contrasting responses of plant Na^+^ fluxes to Si.** In pre-labeled roots of intact rice seedlings, ^24^Na^+^ eﬄux shows no difference in plants grown with or without Si in the presence of high salinity (main panel; redrawn from Malagoli et al., 2008). By contrast, Na^+^ fluxes from root to shoot are highly sensitive to Si supply (inset; redrawn from Gong et al., 2006).

Limiting shoot Na^+^ and Cl^-^ accumulation is critical to salt tolerance in many species, as it may prevent leaf metabolic disorders, ion imbalances, and the desiccation of leaf tissue via osmotic stress (Oertli, 1968; Flowers et al., 1991; Kronzucker et al., 2013). This is particularly important in rice, where, in addition to normal transpiration, involving xylem loading via the symplast, there is a pronounced transpirational bypass flow, *i.e.*, a bypassing of the symplast in regions where endodermal barriers are underdeveloped or absent (root tips, or zones of lateral root emergence; Yeo et al., 1999; Gong et al., 2006; Shi et al., 2013; see also Speer and Kaiser, 1991; Flowers, 2004; Saqib et al., 2008; Coskun et al., 2013a; Shazad et al., 2013). Si provision has been shown to reduce root-to-shoot translocation of both Na^+^ and Cl^-^ in salt-stressed rice, despite increasing transpiration and stomatal conductance, indicating that Si does not act to reduce sodium translocation by reducing transpiration *per se*, but rather by blocking bypass flow (Yeo et al., 1999; Gong et al., 2006; **Figure 1**, inset). The proposal that Si deposition in endodermal and exodermal Casparian bands forms physical barriers to Na^+^ and Cl^-^ translocation is supported by X-ray localization patterns of Si deposition, and the greatly reduced translocation, with Si treatment, of the apoplastic dye trisodium-8-hydroxy-1,3,6-pyrenetrisulphonic acid (PTS) (Gong et al., 2006; Shi et al., 2013; see also Lux et al., 2003). Whether this mechanism is peculiar to rice, or is taxonomically widespread, awaits investigation in other species.

Effects of Si on transpiration depend on species and environmental conditions. While Si increased transpiration in both drought and salt-stressed rice (Chen et al., 2011; see above), Si decreased transpiration in non-stressed rice (Ma and Takahashi, 1993; Agarie et al., 1998). Similar observations were found in drought-stressed wheat (Gong et al., 2005) and sorghum (Hattori et al., 2005; Ahmed et al., 2011), while, by contrast, Si reduced transpiration in drought-stressed maize (Gao et al., 2004, 2006), and had no effect in cucumber (Hattori et al., 2008). Such variability suggests divergent strategies among species, as they balance rates of water uptake and those of leaf-level water loss. The mechanisms underlying these strategies and responses to Si require much more discovery and analysis.

## Water Transport and Plant Water Status

Salt- and drought-stressed plants have reduced water uptake and content, both of which can be alleviated by Si provision, which leads to improved water status and water-use efficiency in many species (Gao et al., 2004; Liu et al., 2014; Wang et al., 2015; Zhu et al., 2015; Shi et al., 2016). In sorghum, for example, Si increased root and whole-plant hydraulic conductance, transpiration, stomatal conductance, and leaf water content under osmotic stress (Liu et al., 2014, 2015). The increase in root hydraulic conductance coincided with a 2- to 4-fold increased expression of plasma-membrane intrinsic protein (PIP) aquaporins, and increased PIP-mediated water transport was suggested by inhibition of the water flux by mercury (Hg^2+^) (Liu et al., 2014, 2015; see also Zhu et al., 2015). The use of Hg^2+^ as an aquaporin inhibitor should be taken with caution, however, as it can also inhibit influx of K^+^ (Coskun et al., 2012), which can affect water transport due to the important osmotic role of K^+^ (Dolan and Davies, 2004). Regardless, the mechanisms by which Si nutrition affects aquaporin expression and activity have yet to be resolved.

It is interesting that Si transport is also mediated by aquaporins, specifically members of the Nod26-like major intrinsic protein (NIP) III subgroup (Ma and Yamaji, 2015). The expression pattern of *Lsi1*, a NIP homolog encoding a Si influx transporter, shows varying responses to Si nutrition in different species. For example, its expression in response to Si provision is downregulated in rice and soybean, unaffected in maize, barley, and wheat, and upregulated in cucumber (Ma and Yamaji, 2015; and references therein). Interestingly, under salinity stress, *OsLsi1* expression was upregulated in roots of both a salt-sensitive and -tolerant variety of rice (1.82- and 2.12-fold, respectively; Senadheera et al., 2009). The authors proposed that this may result in greater Si uptake in the salt-tolerant variety compared to the salt-sensitive one, with enhanced Si deposition in the transpirational bypass route, and thus restricted shoot Na^+^ translocation. Given that rice *OsLsi1* expression shows opposing responses to Si supply and salinity stress separately, it would be interesting to see how expression responds to co-application in this important, salt-sensitive, and highly water-demanding species.

Besides affecting hydraulic conductance and water transport by modulating aquaporin expression/activity, Si can affect water transport by adjusting the osmotic potential of cells through increased osmolyte accumulation (*e.g.*, proline, soluble sugars, inorganic ions, etc.; Pei et al., 2010; Sonobe et al., 2010; Ming et al., 2012; Liu et al., 2014). Increased root hydraulic conductance may also be attributed to Si-induced reductions in oxidative stress and membrane damage (Shi et al., 2016; see below).

## Ion Transport

The reduction of Na^+^ influx from the external solution into the cytosol, and the increase of Na^+^ eﬄux in the opposite direction (or from cytosol to vacuole) have been proposed to be major salt-tolerance mechanisms; both work toward lowering cytosolic Na^+^ pools (Munns and Tester, 2008). In addition, homeostatic maintenance of intracellular K^+^ pools under salt stress is critical to maintain proper cell function (Kronzucker et al., 2013). Si may alleviate salinity stress by influencing these aspects of Na^+^ and K^+^ transport and accumulation (for review, see Zhu and Gong, 2014; Rizwan et al., 2015). In salt-stressed barley, activities of root plasma membrane H^+^-ATPase, and tonoplast H^+^-ATPase and H^+^-PPase, are stimulated under Si supply (Liang, 1999; Liang et al., 2005, 2006). These changes in cellular H^+^ pumps have been proposed to enhance Na^+^ eﬄux via the Na^+^-H^+^ exchangers HvSOS1 and HvNHX1 (in the plasma membrane and tonoplast, respectively), and K^+^ influx via K^+^-H^+^ symporters such as HvHAK1, as they are secondarily active fluxes driven by electrochemical H^+^ gradients (Liang et al., 2005, 2006). However, proton-pump stimulation may be indirect, as H^+^-ATPase activity was unaffected by Si in plasma membrane vesicles from leaves of salt-stressed barley (Liang et al., 2006). Moreover, we have found no evidence for an effect of Si on putatively SOS1-mediated Na^+^ eﬄux in our own laboratory, in roots of intact rice seedlings under salinity stress (**Figure 1**, main panel; see also Malagoli et al., 2008). Nevertheless, this is an interesting potential mechanism of Si-mediated salt tolerance that requires further investigation by various means, including measurements of Na^+^ fluxes in root tips, where recent physiological evidence of Na^+^-H^+^ antiport activity has been demonstrated (Hamam et al., 2016), as well as *in planta* K^+^ fluxes (Coskun et al., 2014).

Silicon can stimulate synthesis and accumulation of polyamines (PA) such as putrescine, spermidine, and spermine, in salt-stressed plants, and this has also been proposed to help mediate salt tolerance (Gill and Tuteja, 2010a; Wang et al., 2015; Yin et al., 2016; see also below). PA production may function in this way via regulating K^+^ and Na^+^ transport, improving antioxidant ability, and modifying osmotic potential (Kusano et al., 2008; Alcazar et al., 2010). Patch-clamp analysis in root epidermal and cortical protoplasts from salt-stressed barley showed that PAs blocked inward and outward Na^+^ and K^+^ currents via non-selective cation channels (NSCCs; Zhao et al., 2007), suggesting that this may prevent toxic intracellular accumulation of Na^+^; however, it is important to note that *in planta* evidence of NSCC-mediated fluxes is currently lacking (Kronzucker and Britto, 2011; Coskun et al., 2013b), and such claims should be interpreted cautiously.

## Oxidative Stress

Lipid peroxidation by reactive oxygen species (ROS) is another major mechanism of salt toxicity in higher plants (Hernandez et al., 1993; Fadzilla et al., 1997; Zhu, 2001; Gill and Tuteja, 2010b). Si has been shown to decrease the concentration of malondialdehyde (MDA), the end-product of lipid peroxidation, in salt-stressed barley (Liang et al., 2003), maize (Moussa, 2006), and grapevine rootstock (Soylemezoglu et al., 2009), and thus may help to maintain membrane integrity and decrease permeability (Liang et al., 2015). Si has also been shown to increase the activity of key antioxidant defense enzymes superoxide dismutase (SOD), peroxidase (POD), catalase (CAT), as well as glutathione reductase (GR) activities and the glutathione (GSH) concentration in salt-stressed plants (Liang et al., 2003, 2006; Al-Aghabary et al., 2004; Zhu et al., 2004; Shi et al., 2016). Khoshgoftarmanesh et al. (2014) showed that MDA concentrations were positively correlated with Na^+^ uptake in salt-stressed cucumber but negatively correlated with Ca^2+^ and K^+^ uptake, and with Si supply. How Si mediates this response is unclear, but the explanation that, under Si supply, stabilized membranes lead to symplastic [Na^+^] reductions, and [K^+^] and [Ca^2+^] increases, is more parsimonious than one invoking altered ion transporters such as NSCCs (see above).

## Si Fertilization and Agricultural Gains

Silicon is the second most abundant soil element after oxygen, comprising ∼29% of the Earth’s crust (Haynes, 2014). This is mostly as insoluble crystalline aluminosilicates, which are not plant-available. Soluble, bioavailable Si, by contrast (*i.e.*, monosilicic/orthosilicic acid; H_4_SiO_4_), normally ranges between 0.1 and 0.6 mM in soils (Epstein, 1994). H_4_SiO_4_ is weakly acidic (*pKa*_1_ = 9.84, *pKa*_2_ = 13.2), and thus is largely undissociated in most soils. The traditional view, that bioavailable Si derives from solvation of primary and secondary minerals and buffered by the adsorption and desorption of silicate onto sesquioxides, has been supplanted by the idea that phytogenic cycling of Si (uptake by plants, silica formation mainly in leaves, and return to the soil as plant litter) is the main determinant of bioavailable soil Si in natural ecosystems (Haynes, 2014; see also Pii et al., 2015; Gattullo et al., 2016). Si pools in agricultural soils are often low due to the regular removal of Si-rich litter during harvest, a practice which may be altering terrestrial and global Si cycling (Savant et al., 1997; Struyf et al., 2010; Vandevenne et al., 2015).

Use of Si fertilizers began in the 1950s in Japan and is now widespread (Guntzer et al., 2012), the most common sources being industrial slags (Haynes, 2014), as well as plant straw, typically from rice (Hossain et al., 2001). These applications have been effective in enhancing the yield and quality of many agricultural crops, including both monocots such as rice, wheat, maize, barley, millet, sorghum, and sugarcane, and dicots such as cotton and soybean (Liang et al., 2015; and references therein).

It has been claimed that Si primarily benefits stressed plants, with minor effects on unstressed plants (Fauteux et al., 2006; Chain et al., 2009; Epstein, 2009; Van Bockhaven et al., 2013). For example, Si addition showed little alteration of the transcriptome of unstressed *Arabidopsis* (Fauteux et al., 2006), wheat (Chain et al., 2009), and rice (Watanabe et al., 2004; *cf*. Brunings et al., 2009; Van Bockhaven et al., 2013), and the proteome of rice (Nwugo and Huerta, 2011). However, such changes do not necessarily indicate the lack of a beneficial role. In long-term studies, Si was shown to increase crop yields, even in unstressed rice; this was attributed to lower transpiration by spikelets (Tamai and Ma, 2008; Detmann et al., 2012, 2013). Si was also shown to increase yields of rice growing under non-stressed conditions by altering source-sink relationships and increasing photosynthesis, mesophyll conductance, N-use efficiency, and mobilization of photoassimilates and amino acids from vegetative tissues to grains (Detmann et al., 2012, 2013). The authors suggested that Si may act as a signaling factor redirecting the primary metabolism of plants, although the mechanism by which this is achieved is not known; this is an exciting and promising new avenue of Si research.

Lastly, a new prospect of Si research involves “seed priming,” whereby exposing seeds to Si for only a few hours fortifies plants against future stress events. Currently, the priming literature is focused on biotic stress resistance (van Hulten et al., 2006; Conrath, 2011; Van Bockhaven et al., 2013), and relatively few studies have investigated effects on performance under abiotic stress; however, several interesting and promising findings have emerged. For example, wheat seeds treated with Si for 6–8 h showed significant increases in germination rate, vegetative growth, and crop yield under salinity and osmotic stresses, compared to non-primed seeds (Hameed et al., 2013; Azeem et al., 2015; Ahmed et al., 2016). Similarly, maize plants grown from seeds treated for 12 h in Si showed increased growth, leaf relative water content, and levels of photosynthetic pigments, soluble sugars, soluble proteins, total free amino acids, potassium, and activities of SOD, CAT, and POD enzymes, compared to plants that were not primed (Latef and Tran, 2016). Moreover, Si-primed seedlings showed decreased proline, MDA, and Na^+^ contents. Although the underlying mechanisms are unknown, and much more investigation is required, seed priming appears to be a promising and cost-effective procedure to confer resistance to major stresses such as drought and salinity.

## Conclusion

Plant silicon research has made great strides since the designation of Si as “non-essential.” While this element is still generally excluded from most plant growth-media formulations, it is now widely accepted to benefit many plant species, including many agriculturally prominent crops. While these benefits may be particularly pronounced under stresses such as drought and salinity, growing evidence indicates that Si may also improve growth under relatively benign conditions. Today, the multiple threats faced by our species, including rapid human population growth, changing climate, and increasing salinity, add urgency to the investigation of crop improvement by Si.

## Author Contributions

DC wrote the manuscript, with input and editing from DB, WH, and HK.

## Conflict of Interest Statement

The authors declare that the research was conducted in the absence of any commercial or financial relationships that could be construed as a potential conflict of interest.
